# Treatment outcome of the implementation of HIV test and treat policy at The AIDs Support Organization (TASO) Tororo clinic, Eastern Uganda: A retrospective cohort study

**DOI:** 10.1371/journal.pone.0239087

**Published:** 2020-09-22

**Authors:** Ronald Opito, Joseph Mpagi, Denis Bwayo, Francis Okello, Kenneth Mugisha, Agnes Napyo

**Affiliations:** 1 Department of Public Health, Busitema University, Mbale, Uganda; 2 Directorate of Program Management and Capacity Development, The AIDs Support Organization (TASO), Kampala, Uganda; 3 Department of Microbiology, Busitema University, Mbale, Uganda; 4 Department of Internal Medicine, Busitema University, Mbale, Uganda; University of Ghana College of Health Sciences, GHANA

## Abstract

**Background:**

Uganda has been making progress towards universal HIV test and treat since 2013 and the 2016 test and treat policy was expanded from the 2013 guidelines. The expanded policy was rolled out in 2017 across the country. The treatment outcomes of this new policy have not yet been assessed at program level. The objective of this study was to determine the treatment outcome of the HIV test and treat policy in TASO Tororo Clinic, Eastern Uganda.

**Methodology:**

This was a retrospective cohort study using secondary data. The study involved 580 clients who were newly diagnosed HIV positive in TASO Tororo clinic between June 2017 and May 2018, who were then followed up for ART initiation, retention in care, viral load monitoring and viral load suppression. The data was analyzed using Stat 14.0 version statistical software application.

**Results:**

Of the 580 clients, 93.1%(540) were adults aged ≥20 years. The uptake of test and treat was at 92.4%(536) and 12 months retention was at 78.7% (422). The factors associated with retention in care were a) being counselled before ART initiation, AOR 2.41 (95%CI, 1.56–3.71), b) having a treatment supporter, AOR 1.57 (95%CI, 1.02–2.43) and having an opportunistic infection, AOR 2.99 (95%CI:1.21–7.41). The viral load coverage was 52.4% (221) and viral load suppression rate was 89.1% (197) of clients monitored. Age <20 years was the only identified factor associated with vial load non suppression, AOR 7.35 (95% CI = 2.23–24.24).

**Conclusion:**

This study found high uptake of ART under test and treat policy, with very low viral load coverage, and a high viral load suppression rate among those monitored. The study therefore highlights a need to differentiate viral load testing based on the population needs and ensure each client testing positive receives pre-ART initiation counselling so as to improve retention in care.

## Introduction

HIV is still a global burden despite the efforts made to combat it and as per the UNAIDS 2019 report, there are 37.9million people living with the disease globally, with 1.7million new HIV infections and 770,000 AIDS-related deaths [1]. This burden is however skewed towards sub-Saharan Africa, with 61% of the new infections and 68% of the 37.9million people living in the sub Saharan Africa [1].

Uganda contributes 1.4million people living with HIV to the global burden [1], with a national prevalence of 6.2% [2]. HIV prevalence in Mid-Eastern Uganda where TASO Tororo clinic is located was estimated at 5.1% [2]. Based on the Uganda Bureau of Statistics (UBOS) 2014 census report released in 2016, Tororo district had a total population of 517,082 people [3]. The total number of individuals living with HIV in the district was therefore estimated to be 26,372, of which by end of Dec 2018, 16,544 (62.5%) were on care and 8066 (48.75%) were receiving ART from TASO Tororo clinic.

With the high burden of HIV in Uganda, the country has been making progress towards universal HIV treatment since 2013 when it adopted new WHO recommendations. This 2013 guideline expanded eligibility to ART to include test and treat, regardless of WHO clinical stage or CD4 level for all pregnant and lactating women which was started under Option B^+^, all children under 15years, HIV positive clients in a sero-discordant relationship, HIV positive clients in the category of key and priority populations, and expanded the CD4 cut off for ART initiation for all the other categories to 500 cells/mm^3^, up from the 350 cut off [4]. The current (2016) test and treat policy was expanded from the 2013 guidelines and involves providing lifelong ART to all people living with HIV irrespective of CD4 level or HIV clinical stage. It was rolled out in 2017 across the country in a stepwise manner [5].

Studies have demonstrated that prompt ART initiation regardless of CD4 counts or clinical stage is associated with better treatment outcomes and there has been a global shift towards universal test and treat intervention. The uptake of test and treat among children in Uganda was found to be 49.1% with better outcomes among those who initiated ART within seven days reported as death at 8.3% and LTFU at 3.3% compared to 14. 4% and 8.6% respectively in the later initiators [6]. Whereas 28% of 343 women at high risk for HIV infection in Kampala-Uganda initiated ART promptly, with sex work as main job, younger age and being widowed/separated found to be associated with lower odds of prompt ART [7]. Studies on test and treat programmatic outcome in Zimbabwe found same day initiation on ART among 972 newly identified positives to be 65% and 90% of patients initiated on ART were retained in care at 3 months [8].

Barriers to starting ART under test and treat have included cost, poor interactions with Health care workers, feeling healthy, concerns about ART side effects; fear of HIV disclosure and discrimination, limited privacy at health facilities; and fear of long waiting times [9, 10]. Men’s social influence, masculine feelings of strength, and success with their sexual partners led them to delay treatment until they were sick [11]. On the other hand, facilitators to uptake of ART identified include, the urge to stay healthy and to take care of children/dependents, clients’ tailored counselling services, excellent quality of health education, good patient flow, HIV status disclosure to partner and integrated HIV/primary care services [9, 12].

Earlier studies on ART treatment indicated that there were some poor outcomes among those initiating ART under set criteria as CD4 and WHO clinical stage. These poor outcomes reported include, high death and lost to follow up (LTFU) rates among those initiating ART [13, 14], delayed ART initiation [6, 7], inadequate adherence to ART [15, 16] and below target levels of viral load suppression [6, 15, 17]. In Uganda specifically, the viral load suppression has remained at 56.0%, much less than the UNAIDS2020 target of 90%, amidst the various interventions put in place, including test and treat [1].

Routine viral load monitoring improves HIV treatment outcome and helps in understanding of HIV surveillance and epidemic trends. It provides data on HIV service coverage and quality. At community level it provides information on HIV transmission risk [18]. Access to routine viral load monitoring is critical to ensuring treatment success but this remains limited in low- and middle-income countries [19, 20].

There are limited studies conducted on the treatment outcomes of the new policy in Uganda. One study which compared treatment outcomes among children initiated on treatment promptly (within 1 week of HIV diagnosis) and those that delayed treatment; found shorter time to viral suppression among prompt ART initiators than those who delayed (24.9months vs 38.5months) [6]. Another study assessed the factors associated with prompt ART initiation among women with high risk of HIV infection in Kampala and found low uptake (28%) of test and treat intervention [7]. No similar studies have been conducted in Tororo district and this study addressed the gap.

This study explored the outcomes of the implementation of HIV/AIDS test and treat policy in The AIDs Support Organization (TASO) Tororo clinic, Tororo district, Eastern Uganda.

## Materials and methods

### Study area

The study was conducted in TASO Tororo clinic, one of the 11 TASO Centers in Uganda. The center was founded in 1991 in Tororo district, and currently located in the Eastern Division, COX road of Tororo Municipality Tororo district. By December 2018, it was providing care to 8066 people living with HIV with a catchment area of 75KM radius, and had identified 1130 new HIV positive individuals between June 2017 and May 2018. These individuals were identified and initiated on ART during both facility and community outreach HIV testing by HTS volunteers. The 8066 PLHIV provided with ART at TASO Tororo clinic made up 48.75% of all clients (16,544) receiving ART in Tororo district. Besides HIV/AIDS, TB/HIV prevention and treatment services, TASO Tororo provides a comprehensive package such as community sensitization on HIV testing and prevention services, screening and treatment of opportunistic infections (OIs), sexual reproductive health services (SRHs), gender based violence (GBV), and Family Health services such as Nutrition Assessment and Counseling services (NACS), screening and treatment for malaria, maternal neonatal and child health (MNCH) to the HIV positive clients and their exposed children.

Tororo district is located about 220km East of Uganda’s Capital city, Kampala and boarders Western Kenya. There are two main urban centres in Tororo, that is Tororo Municipality and Malaba Town council (boarders Kenya), with a number of factories and construction work, thereby making the population very mobile within these town areas, though relatively stable in the rural areas.

### Study population and sample size determination

The study population were all patients who were newly diagnosed HIV positive at TASO Tororo clinic between June 2017 and May 2018. The sample size of 422 was determined using the Cochran equation. However, all the eligible study participants (580) were included in the data analysis.

### Study design and data collection procedure

The study used a retrospective cohort design using secondary data. We applied quantitative method of data collection and analysis. We used data abstraction tool designed based on the HIV Testing Services (HTS) register, HIV care card and electronic medical record system which are used for routine monitoring of linkage to care, ART initiation and follow up. A retrospective medical record review was selected because the data required to answer the study objectives was available within the routinely collected data.

The data abstraction involved patients who were newly diagnosed HIV positive at TASO Tororo between June 2017 and May 2018 who were followed up from HTS register, to ART register and electronic medical records.

### Inclusion and exclusion criteria

The people included in the study were HIV positive clients newly diagnosed at TASO Tororo between June 2017 and May 2018 and those with complete information. While those excluded were clients with incomplete information such as age, sex and date of HIV diagnosis and who missed to be entered into the HTS (HIV Testing Services) register.

### Study vairables

The main outcome was the uptake of ART under the test and treat policy. This was assessed by determining the number of clients testing positive within the study period and the proportions of those who were initiated on ART within one month of HIV diagnosis. The secondary outcomes assessed were; twelve months retention into care assessed by determining the proportions of clients who were still active in TASO Tororo care 12 months after initiation under test and treat policy, viral load monitoring assessed by the presence of viral load result slip in the client’s file or captured in the electronic medical records done within a 12 month period and viral load suppression measured within 12 months of ART initiation. Plasma Viral loads measurement at TASO Tororo was done by Central Public Health Laboratories (CPHL) through a hub system and a patient with a suppressed viral load was considered as one with RNA Viral load <1000copies/mL.

Independent variables included age categorized as <20 years (children and adolescents) or ≥20years (adults), sex, baseline WHO clinical stage, educational level, Counselling before ART initiation assessed by documenting a counselling session on the ART care card on the day of ART initiation (counselled or not counselled), ART regimen at initiation measured as Efavirenze (EFV) based, Nevirapine (NVP) based or Protease Inhibitors (PI) based regimens, nutritional status measured using mid upper arm circumference (MUAC), and reported using color coded tape as Green (Well nourished), yellow (Moderate malnutrition) and red (severe malnutrition) marital status reported as never married/single, married/cohabiting, divorced/separated and widowed, presence of treatment supporter, residence of the client reported as urban for clients residing within the municipality and rural for clients outside the municipality.

### Data management and analysis

**Fig 1:**
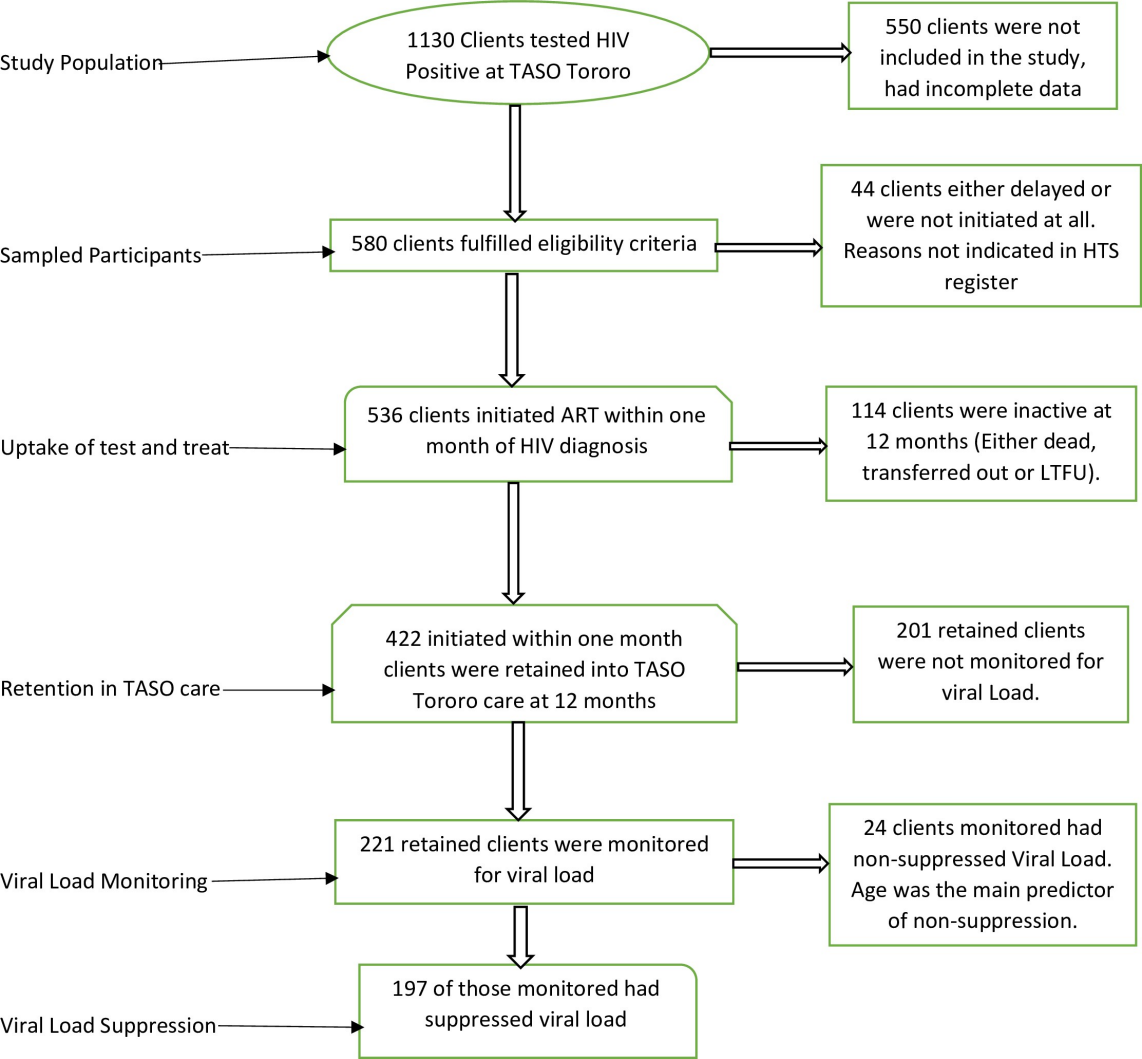
Data analysis flow chart.

The data extracted from HTS register and electronic medical records was entered into excel, merged and then imported to Stata 14.0 statistical software package for further cleaning of missing/mismatching information and coding before analysis. Each study outcome of interest was analyzed separately to determine the associations between the independent and dependent variables. The analysis was done in a stepwise manner, whereby after the first outcome (uptake of test and treat), clients who delayed treatment were dropped while analyzing the 2^nd^ outcome (retention) and those who were inactive were dropped while analyzing viral load coverage, and clients who were not monitored for viral load were dropped while analyzing viral load non-suppression (Fig 1).

Quality assurance and validity of data was enhanced through training of research assistants on standardized data collection procedures and strict supervision of data collection and entry.

Univariate analysis was done for socio-demographic and baseline clinical characteristics and was presented in form of frequencies, proportions and means. Binary logistic regression was used to determine the association between binary outcomes and the independent variables. Multivariate logistic regression was used to assess the factors associated with uptake of ART under test and treat policy, retention, viral load monitoring and suppression at multivariate level. Variables were selected for inclusion into the multivariate models based on their levels of significance at bivariate level(P≤0.25) and on their biological plausibility to the outcomes of interest. Significance level was set at 5%.

### Ethical consideration

The study protocol was reviewed and approved by CURE Children’s Hospital of Uganda Research & Ethics Committee (CCHU-REC/16/019), which also granted consent waiver for this study, given that it was utilizing already collected program data and there was no direct contact with the clients. Permission to utilize TASO Tororo program data was sought from the Executive Director TASO Uganda Limited.

## Results

### Participant baseline characteristics, uptake and retention into care

There were 580 individuals who were diagnosed HIV positive in TASO Tororo within the study period, found to have complete data and were included in the analysis. Their mean (SD) age was 38.0 ± 13.1 years; 56.5% (n = 328) were females and 93.1% (n = 540) were adults aged ≥20 years, 89.8%(n = 521) of the identified HIV positive were from the community outreach testing site and 61.7% (n = 358) were married/cohabiting (Table 1).

**Table 1:** Baseline characteristics of study participants and factors associated with failure take up test and treat policy.

Participant Characteristic	Proportions, N(%)	No of clients who took up test and treat, n(%),	No of clients who did not take up test and treat n(%)	UOR (95% CI)^a^	AOR (95%CI)^b^
	Total (N = 580)	n = 536	n = 44		
Sex,					
Male	252 (43.5)	228 (90.5)	24 (9.5)	1.62 (0.87–3.01)	-
Female	328 (56.5)	308 (93.9)	20 (6.1)	1.00	-
Age/years					
<20 (Child. & Adol.)	40 (6.9)	37 (92.5)	3 (7.5)	1.00	1.00
≥20 (Adults)	540 (93.1)	499 (92.4)	41 (7.6)	1.01 (0.30–3.43)	2.48 (0.46–13.34)
Age in years, Mean (SD)	38.0 (13.1)				
Education					
None	148 (25.5)	141 (95.3)	7 (4.7)	1.00	1.00
Primary	343 (59.1)	318 (92.7)	25 (7.3)	1.58 (0.67–3.75)	1.78 (0.73–4.35)
Post-Primary	89 (15.3)	77 (86.5)	12 (13.5)	3.14 (1.19–8.30)*	2.76 (0.95–7.69)
Marital Status					
Divorced/Separated/Widowed	169 (29.1)	157 (92.9)	12 (7.1)	1.00	1.00
Married/Cohabiting	358 (61.7)	333 (93.0)	25 (7.0)	0.98 (0.48–2.01)	1.04 (0.50–2.14)
Never married	53 (9.1)	46 (86.8)	7 (13.2)	1.99 (0.74–5.35)	3.53 (0.95–13.15)
Residence					
Rural	326 (56.2)	305 (93.6)	21 (6.4)	1.00	1.00
Urban	254 (43.8)	231 (90.9)	23 (9.1)	1.45 (0.78–2.68)	1.39 (0.74–2.16)
Care Entry Point					
Facility	59 (10.2)	49 (83.0)	10 (17.0)	1.00	1.00
Outreach	521 (89.8)	487 (93.5)	34 (6.5)	0.41 (0.19–0.92)*	0.36 (0.16–0.79)*
Previously Tested					
No	302 (42.3)	286 (94.7)	16 (5.3)	1.00	-
Yes	278 (47.7)	250 (89.9)	28 (10.1)	0.88–3.28)	-

*Bold = significant with P-Value<0.05

^a^UOR = Unadjusted odds ratio.

^b^AOR = Adjusted odds ratio, (Child. & Adol.) = Children and Adolescents.

Of the 580 clients tested positive within this period, 92.4% (n = 536, 95% CI = 90.0–94.3), were initiated on ART under test and treat policy. The reasons for failure to initiate on ART within one month of HIV diagnosis were not indicated in the HTS register, but being tested positive at the outreach site was associated with lower odds of failure to take up test and treat, AOR of 0.36 (95%CI, 0.16–0.79) as compared to those tested at the facility.

Twelve month retention for the clients who initiated ART under test and treat policy at TASO Tororo ART program was at 78.7%(n = 422, 95% CI = 75.0–82.0).

### Factors associated with retention into TASO Tororo care at 12 months of enrollment on test and treat

Of the 536 clients who initiated ART under test and treat policy, 78.7% (n = 422, 95% CI = 75.0–82.0) were active in TASO Tororo care by 12 months of ART initiation. The 21.3% (n = 114, 95% CI = 18.0–25.0) of the clients who were no longer active in TASO Tororo care were either transferred out to other health facilities, 7.3% (n = 39), dead 1.3% (n = 7) or lost to follow up 12.7% (n = 68).

The factors positively associated with retention in TASO Tororo care were a) being counselled before ART initiation with AOR of 2.41 (95%CI, 1.56–3.71), b) availability of treatment supporter at the time of ART initiation with AOR of 1.57 (95%CI, 1.02–2.43) and having an opportunistic infection at the time of ART initiation with AOR of 2.99 (1.21–7.41). Table 2 for details.

**Table 2:** Factors associated with retention into TASO Tororo care at 12 months of enrollment on test and treat.

Factors.	Total Clients initiated test and treat	Active at 12months, n (%)	Inactive at 12 months, n (%)	UOR (95%CI)	AOR (95%CI)
	N = 536	n = 422	n = 114		
Sex,					
Female	308	246 (79.9)	62 (20.1)	1.00	1.00
Male	228	176 (77.2)	52 (22.8)	0.85 (0.56–1.29)	0.85 (0.55–1.31)
Age/years					
<20 (Child. & Adol.)	37	30 (81.1)	7 (18.9)	1.00	1.00
≥20 (Adults)	499	392 (78.6)	107 (21.4)	0.85 (0.37–2.00)	0.88 (0.36–2.15)
Education					
None	141	107 (75.9)	34 (24.1)	1.00	1.00
Primary	318	248 (78.0)	70 (22.0)	1.13 (0.70–1.80)	1.04 (0.63–1.72)
Post-Primary	77	67 (87.0)	10 (13.0)	2.13 (0.99–4.59)	2.21 (0.98–4.98)
Residence					
Rural	305	214 (70.2)	91 (29.8)	1.00	1.00
Urban	231	180 (77.9)	51 (22.1)	0.92 (0.61–1.39)	0.96 (0.62–1.48)
B/L N/Status (MUAC)					
Green	501	395 (78.8)	106 (21.2)	1.00	1.00
Red/Yellow	35	27 (77.1)	8 (22.9)	0.91 (0.40–2.05)	0.82 (0.35–1.95)
Counselled at ART Initiation					
No	220	155 (70.5)	65 (29.5)	1.00	1.00
Yes	316	267 (84.5)	49 (15.5)	2.29 (1.50–3.48)*	2.41 (1.56–3.71)*
Availability of T/Supporter					
No	221	165 (74.7)	56 (25.3)	1.00	1.00
Yes	315	258 (81.9)	57 (18.1)	1.57 (1.04–2.38)*	1.57 (1.02–2.43)*
Presence of O. Is					
No	477	369(77.4)	108(22.6)	1.00	1.00
Yes	59	53 (89.8)	6 (10.2)	2.59(1.08–6.18)*	2.99 (1.21–7.41)*

*Bold = significant with P-Value <0.05, UOR = Unadjusted Odds Ratio, AOR = Adjusted Odds Ratio, T/supporter = treatment supporter, O.Is = Opportunistic Infections. B/L N/Status = baseline nutritional status.

### Factors associated with failure to monitor active clients for viral load

Of the 422 clients who were active in TASO Tororo care at 12 months, 52.4% (n = 221, 95%CI = 47.6–57.1) were monitored for Viral load and 47.6% (n = 201, 95% CI = 42.9–52.4) were not monitored. The odds of failure to monitor for viral load was lower among clients living in an urban area, AOR 0.46 (95% CI, 0.21–0.68). The detail is shown in Table 3 below.

**Table 3:** Showing factors associated with failure to monitor for viral load.

Factors	No of Clients Active in care at 12 Months, N	No of clients Monitored for Viral Load, n (%)	No of clients Not Monitored for VL, n (%)	UOR(95%CI)	AOR(95%CI)
	N = 422	n = 211	n = 201		
Age/years					
<20 (Child. & Adol.)	30	22 (73.3)	8 (26.7)	1.00	1.00
≥20 (Adults)	392	199 (50.8)	193 (49.2)	2.67 (1.16–6.13)*	2.30 (0.97–5.43)
Residence					
Urban	180	114 (63.3)	66(36.7)	0.46 (0.31–0.68)*	0.46 (0.31–0.69)*
Rural	242	107 (44.2)	135(55.8)	1.00	1.00
Presence of O.Is					
Yes	53	36 (67.9)	17(32.1)	0.47 (0.25–0.88)*	0.55 (0.29–1.04)
No	369	185 (50.2)	184(49.8)	1.00	1.00
Counselled at ART initiation					
Yes	267	130 (48.7)	137(51.3)	1.50 (1.00–2.23)*	1.41 (0.93–2.14)
No	155	91 (58.7)	64(41.3)	1.00	1.00
Baseline Clinical Stage					
Stage I/II	411	215 (52.3)	196 (47.7)	1.00	-
Stage III/IV	11	6 (54.6)	5 (45.4)	0.91 (0.27–3.04)	-
Baseline TB Status					
No TB	416	217 (52.2)	199 (47.8)	1.83 (0.33–10.12)	-
Has TB	6	4 (66.7)	2 (33.3)	1.00	-
Baseline Nutrition Status (MUAC)					
Green	395	202 (51.1)	193 (48.9)	2.27 (0.97–5.31)	1.96 (0.81–4.73)
Yellow/Red	27	19 (70.4)	8 (29.6)	1.00	

*Bold = Significant with P-Value <0.05, UOR = Unadjusted Odds Ratio, AOR = Adjusted Odds Ratio.

### Viral load non suppression

Among the 221 clients monitored for viral load and were having valid viral load results in their files, 89.1% (n = 197, 95% CI = 84.3–92.4) had suppressed viral load (VL value <1000copies/ml of plasma sample) and 10.9% (n = 24, 95% CI = 7.4–15.5) had non suppressed viral load (VL value ≥1000copies/ml of plasma sample). There was a marked difference in viral load non-suppression rates between adults ≥20 years (7.5%) and children and Adolescents <20 years (40.9%) and this difference was significant (P<0.001). The factor which was significantly associated with Viral load Non-suppression was age <20 years with AOR of 7.35 (2.23–24.24). The factors such as presence of opportunistic infections, having a treatment supporter and being counselled before ART initiation which were positively associated with 12 months retention in care did not have any association with viral load non suppression both at bivariate and multivariate levels. The baseline clinical stage, TB status and nutritional Status were equally not associated with viral load non suppression. The detail is shown in Table 4 above.

**Table 4:** Factors associated with viral load non-suppression.

Factors	Monitored for VL,	Suppressed VL, N = 197,	Non-Suppressed VL, N = 24,	UOR (95%CI)	AOR(95%CI)
	N = 221	n (%)	n (%)		
Age					
<20 (Child. & Adol.)	22	13 (59.1)	9 (40.9)	8.49 (3.13–23.08)*	7.35 (2.23–24.24)*
≥20 (Adults)	199	184 (92.5)	15 (7.5)	1.00	1.00
Education Level					
None	60	49 (81.7)	11(18.3)	1.00	1.00
Primary	123	115 (93.5)	8(6.5)	0.31 (0.12–0.82)*	0.50 (0.16–1.50)
Post-primary	38	33 (86.8)	5(13.2)	0.67 (0.21–2.12)*	1.43 (0.36–5.60)
Presence of O.Is					
No	185	169 (91.4)	16(8.6)	1.00	1.00
Yes	36	28 (77.8)	8(22.2)	3.02 (1.18–7.71)*	1.91 (0.61–6.02)
Baseline Clinical Stage					
Stage I/II	215	193 (89.8)	22(10.2)	1.00	1.00
Stage III/IV	6	4 (66.7)	2 (33.3)	4.39 (0.76–25.33)	2.29 (0.27–19.60)
Baseline TB Status					
No TB	217	194 (89.4)	23(10.6)	0.36 (0.04–3.56)	-
Has TB	4	3 (75.0)	1(25.0)	1.00	-
B/L N/Status (MUAC)					
Yellow/Red	19	17 (89.5)	2(10.5)	1.00	1.00
Green	202	180 (89.1)	22(10.9)	1.04 (0.22–4.80)	1.16 (0.22–6.07)
Counselled at ART Initiation.					
No	91	81(89.0)	10 (11.0)	1.00	1.00
Yes	130	116 (89.2)	14 (10.8)	0.98 (0.41–2.31)	1.22 (0.46–3.25)
Presence of T/Supporter					
No	84	75 (89.3)	9 (10.7)	1.00	1.00
Yes	137	122 (89.1)	15 (10.9)	1.02 (0.43–2.46)	1.00 (0.36–2.72)

*Bold = significant with P-Value<0.05, UOR = Unadjusted Odds Ratio, AOR = Adjusted Odds Ratio, T/Supporter = treatment supporter, B/L N/Status = Baseline nutritional status.

## Discussion

In this study, we found a high uptake of HIV test and treat program where 92.4% of all individuals who were tested HIV positive accepted ART initiation within one month of HIV diagnosis. This therefore demonstrates a significant progress in the uptake of test and treat program in Uganda particularly and Africa at large, from 28% among women at risk of HIV infection in Kampala [7], 49.1%among children in Uganda [6], and 65% in Zimbabwe [8]. This high uptake of test and treat could have resulted from increased sensitization of the community on the benefits of early ART initiation for clients who are HIV positive, and if sustained will lead to attainment of the UNAIDS2030 target of 95-95-95.

In this study, being tested positive at the outreach site was significantly associated with lower odds of failure to take up test and treat. This study finding is contrary to findings from Zimbabwe [8], and could have resulted from TASO’s community HTS intervention where they made deliberate efforts to engage community leaders (expert clients/volunteers, local council I chairpersons and church leaders) into sensitization and mobilization for HIV testing services [21]. This finding therefore confirms that clients tested at their convenient places can comfortably be initiated on ART under the test and treat policy.

Socio-demographic characteristics such as younger age and being widowed/separated were earlier identified in Uganda [7] as being associated with low chances of prompt ART initiation. This was however not the case in our study as both age and marital status did not have any significant association with failure to enroll clients under the test and treat policy.

On 12 month retention in care, this study found suboptimal retention of 78.7% vs expected 95%. This finding is much lower than earlier reported 12 months retention in care in Uganda which was at 90% among adolescents [22] and 87.8% in Virika Hospital [23]. This could be because the earlier studies reported 12 month retention on clients who were started on ART after staying on pre-ART for sometimes as compared to clients who initiated ART under test and treat. However, clients who initiated test and treat in Uganda and Kenya still had higher retention rates of 89.7% and 89.0% in both males and females respectively [24].

In this study, the factors that were significantly associated with 12 month retention into care included a) being counselled before ART initiation, b) having a treatment supporter and c) having an opportunistic infection at the time of ART initiation. It is evident in this study that treatment supporters are able to offer significant support to the newly enrolled clients on ART and keep them active in care for longer than their counterparts who did not have treatment supporters. Also, to note of importance is the presence of opportunistic infections at the time of enrollment which enabled that clients were retained in care. This retention could have been due to the resultant benefit at enrollment on ART which was coupled by treatment and resolution of the opportunistic infections in the individual. Our study finding of HIV counselling before ART initiation having a significant effect on retention into care reaffirms earlier findings in Malawi [12]. Some of the newly identified HIV positive clients missed pre-ART initiation counselling due to the fact that they were identified in the community by the volunteers who did not take counselling as a priority and this severely affected the clients’ retention in care. The other demographic factors such as age and gender which were significantly associated with retention in care in South Africa [13] did not affect retention in care after the implementation of test and treat policy in TASO Tororo.

In this study, residing in an urban area as compared to rural was significantly associated with lower odds of failure to monitor for viral load. Residing in an urban area could have facilitated viral load monitoring due to the proximity to the clinic and convenient access to get their viral load monitored. Most of the clients who were enrolled on ART in TASO Tororo clinic under test and treat policy were residents in rural area, distant from the clinic. In order to achieve adequate viral load suppression, clients who have been on ART for six months or more need to be monitored for viral load and check their viral load status. This study found viral load monitoring for clients who initiated ART under test and treat very low at only 52.4% against the expected 100% monitoring. This finding highlights a key weakness in the implementation of test and treat policy and generates further questions as to why clients are not being monitored for viral load despite being on ART for 12 months and active in care. This finding is however not unique to TASO Tororo test and treat policy as a multi-country study of HIV treatment and monitoring pattern found viral load coverage to be only at 35% [25] and another study in Thailand found coverage to be 53.8% in 2009, which increased to only 79.8% in 2013 with interventions [20].

On a positive side, this study found generally high viral load suppression (89.1%) and low non suppression (10.9%) among clients who were monitored for viral load after initiating ART under test and treat policy. This suppression rate though still suboptimal is higher than initial findings of viral load suppression in East Africa during a cluster randomized community trial of test and treat where after one year on ART, only 72.9% of the newly diagnosed HIV positive had viral load suppression [26]. Despite the good viral load suppression noted among the clients monitored, this finding is inconclusive since only a handful (221 of 580) of clients who tested positive were monitored for viral load. HIV program implementers should therefore differentiate viral load test based on the population needs and HIV testing services so as to reach even the clients who were initiated on ART at the community level.

Among the few clients monitored, the viral load non-suppression rate found in the study was similar to findings in earlier studies where clients were adequately counselled but took long to be initiated on ART. Initial studies reported viral load non suppression as 11% in Uganda [27], 10.3% in South Africa [13] and 11.5% in Ethiopia [28].

This study found age at the time of ART initiation as the only predictor of suppression based on multivariate analysis. This finding of viral load non suppression being higher in children and adolescents is consistent with earlier findings in Uganda where odds of virological non-suppression was decreasing with increasing age, with children aged 0–4 years had AOR 5.3 (95%CI; 4.6–6.1) and young adolescents had AOR of 4.1 (95%CI; 3.7–4.6) registering the highest odds [27] and the suppression rate in children was as low as 52.3% [6]. All these findings point to a bigger challenge of early Virological ART failure in children and adolescents whether they initiate ART promptly or delay. Therefore, interventions geared towards ensuring that children and adolescents are initiated on the optimal regimens and followed closely for proper adherence need to be put in place.

### Study limitations

The study also had limitations. In this study, only 38% (221) of the clients who were tested positive at the facility were monitored for viral load and as such we were unable to decisively determine the viral load suppression rate under the test and treat policy. The study being retrospective in design, we were unable to assess the health system factors which contributed to low viral load coverage as they were not documented in the records. We were also unable to assess the association between baseline CD4 count and viral load outcomes as with the introduction of test and treat, there was inconsistent CD4 testing at the facility.

Since it was a retrospective cohort study basing on data generated from the health facility, there were client records with many missing information which led to only half (580 of 1130) of the clients with complete data being included in the analysis.

## Conclusion

This study found high uptake of ART under test and treat policy, with very low viral load coverage for those initiated, and a high viral load suppression rate among those who were monitored.

The study therefore highlights a need to differentiate viral load testing based on the population needs so as to reach even the clients who initiated ART at community level with viral load services.

There is need to ensure that each client who tests positive receives psychosocial support (counselling) before ART initiation so as to improve retention in care.

There is need to continuously offer psychosocial support to children and adolescents with a focus on ART adherence so as to improve viral load coverage in this age group.

## Supporting information

S1 Dataset (CSV)
